# Evolutionary freedom in the regulation of the conserved itaconate cluster by Ria1 in related Ustilaginaceae

**DOI:** 10.1186/s40694-018-0058-1

**Published:** 2018-07-28

**Authors:** Elena Geiser, Hamed Hosseinpour Tehrani, Svenja Meyer, Lars M. Blank, Nick Wierckx

**Affiliations:** 1 iAMB – Institute of Applied Microbiology, ABBt – Aachen Biology and Biotechnology, RWTH Aachen University, Worringerweg 1, 52074 Aachen, Germany; 2 BioSC, c/o Forschungszentrum Jülich, 52425 Jülich, Germany

**Keywords:** Activation of silent cluster, (*S*)-2-hydroxyparaconate, (*S*)-2-hydroxyparaconic acid, Itaconic acid, Itatartarate, Secondary metabolites, Transcription factors, Basidiomycota, *Ustilago maydis*

## Abstract

**Background:**

Itaconate is getting growing biotechnological significance, due to its use as a platform compound for the production of bio-based polymers, chemicals, and novel fuels. Currently, *Aspergillus terreus* is used for its industrial production. The Ustilaginaceae family of smut fungi, especially *Ustilago maydis*, has gained biotechnological interest, due to its ability to naturally produce this dicarboxylic acid. The unicellular, non-filamentous growth form makes these fungi promising alternative candidates for itaconate production. Itaconate production was also observed in other Ustilaginaceae species such as *U. cynodontis*, *U. xerochloae*, and *U. vetiveriae*. The investigated species and strains varied in a range of 0–8 g L^−1^ itaconate. The genes responsible for itaconate biosynthesis are not known for these strains and therefore not characterized to explain this variability.

**Results:**

Itaconate production of 13 strains from 7 species known as itaconate producers among the family Ustilaginaceae were further characterized. The sequences of the gene cluster for itaconate synthesis were analyzed by a complete genome sequencing and comparison to the annotated itaconate cluster of *U. maydis*. Additionally, the phylogenetic relationship and inter-species transferability of the itaconate cluster transcription factor Ria1 was investigated in detail. Doing so, itaconate production could be activated or enhanced by overexpression of Ria1 originating from a related species, showing their narrow phylogenetic relatedness.

**Conclusion:**

Itaconate production by Ustilaginaceae species can be considerably increased by changing gene cluster regulation by overexpression of the Ria1 protein, thus contributing to the industrial application of these fungi for the biotechnological production of this valuable biomass derived chemical.

**Electronic supplementary material:**

The online version of this article (10.1186/s40694-018-0058-1) contains supplementary material, which is available to authorized users.

## Background

Secondary metabolites are organic, naturally produced, bioactive compounds with a low molecular weight, that are produced by fungi, bacteria, and plants via pathways not belonging to the primary metabolism of this organism [1, 2]. In 2000, a literature survey identified more than 23,000 already discovered secondary metabolites mainly from the fungal kingdom [1, 3]. Closely related species usually produce related compounds and each compound is produced in a highly-narrowed taxonomy [2, 4]. Genes coding for the biosynthesis of secondary metabolites are usually co-localized in a gene cluster with a size of approximately over 10,000 bp depending on the complexity of the metabolite and regions of non-coding base pairs of up to 2000 bp between the coding genes [2, 5, 6]. In cases of polyketide synthases these regions are more extended [7]. These clusters contain genes coding for corresponding biosynthesis enzymes and transporters, regulatory proteins like transcription factors, and optionally modifying enzymes. Secondary metabolite clusters are often controlled by a complex regulatory network [8]. Several levels of regulation exist, which allow the organism to respond to various environmental influences. Transcription of these clusters can be regulated either by specific/narrow-domain or by global/broad-domain transcription factors or regulators or a combination thereof. Alternatively, regulation can be chromatin-mediated by histone acetylation or methylation [8].

**Fig. 1:**
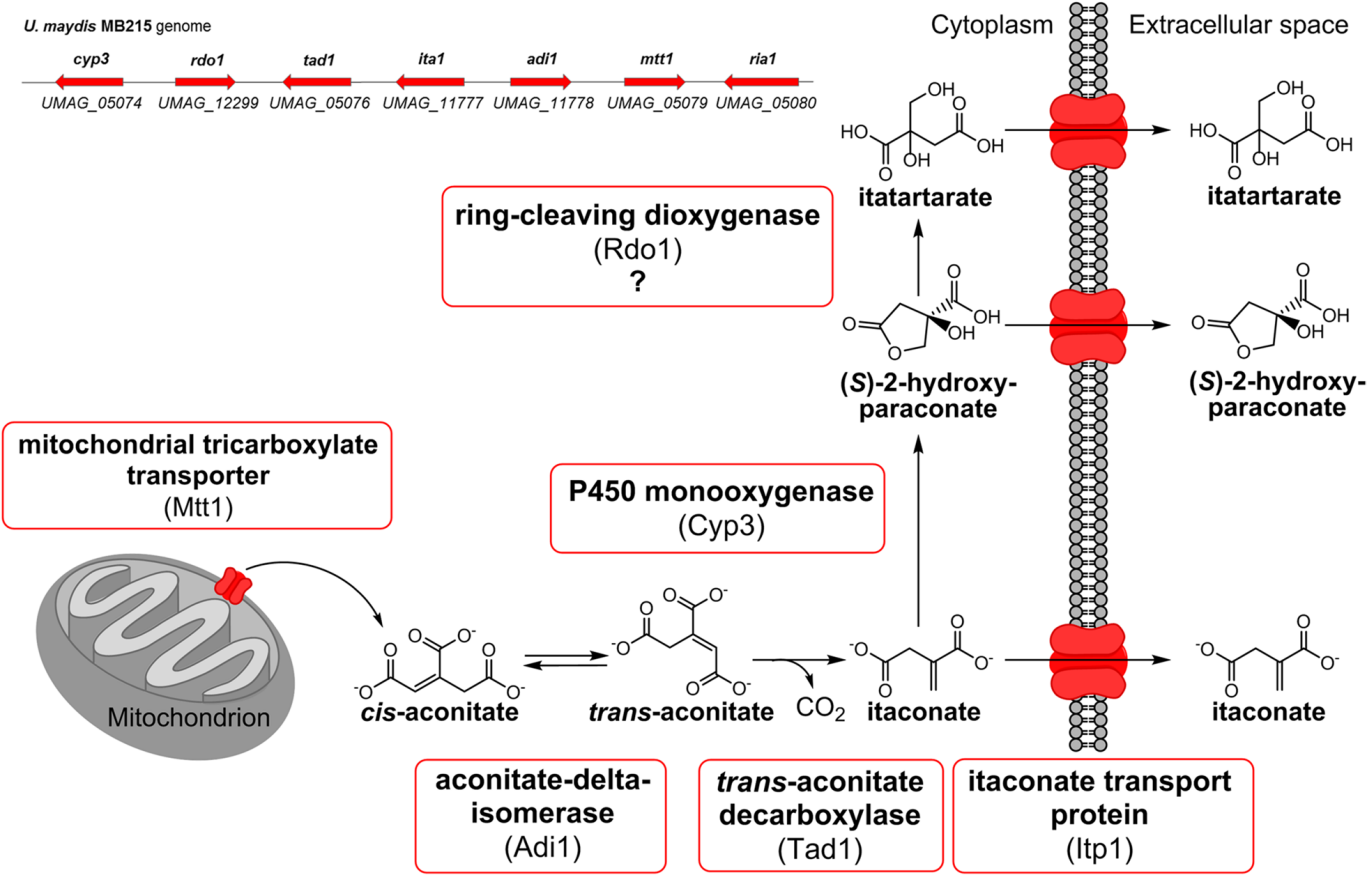
Proposed intracellular organization of the (*S*)-2-hydroxyparaconate biosynthesis pathway in *U. maydis*. *Cis*-aconitate is secreted by the mitochondrial tricarboxylate transporter Mtt1. In the cytosol *cis*-aconitate is converted into itaconate via the intermediate *trans*-aconitate. Itaconate can be further converted to (*S*)-2-hydroxyparaconate by Cyp3. (*S*)-2-hydroxyparaconate might be converted to itatartarate with the help of Rdo1. Secretion of itaconate and possibly (*S*)-2-hydroxyparaconate and itatartarate into the medium is mediated by the major facilitator Itp1. Updated pathway from Geiser et al. [18]

Itaconate and its lactone (*S*)-2-hydroxyparaconate are examples of secondary metabolites. Itaconate is produced by fungi like *Aspergillus terreus* and *Ustilago maydis*, but also by less well-known Ustilaginaceae species, such as *Ustilago cynodontis, Ustilago vetiveriae,* and *Ustilago xerochloae* [9–12]. Itaconate has industrial applications as a co-monomer, for example in the production of acrylonitrile–butadiene–styrene and acrylate latexes in the paper and architectural coating industries [13]. According to an independent evaluation report of the U.S. Department of Energy (DoE) in 2004 [14], itaconate was assigned to be among the top 12 building blocks with a high biotechnological potential, enabling a conversion into a range of new interesting molecules such as 2-or 3-methyltetrahydrofuran with applications as novel biofuels [15, 16]. Recent studies showed that genes for the biosynthesis of itaconate are co-localized in the genome and co-regulated in *U. maydis* [17], and therefore fulfilling the main criteria to be a secondary metabolite. *U. maydis*’ itaconate cluster (GenBank: KT852988.1) contains two itaconate biosynthesis genes *UMAG*_*tad1* and *UMAG*_*adi1* encoding a *trans*-aconitate decarboxylase (Tad1) and an aconitate-Δ-isomerase (Adi1), and two transporter genes *UMAG*_*itp1* and *UMAG*_*mtt1* encoding an itaconate transport protein (Itp1) and a mitochondrial tricarboxylate transporter (Mtt1), respectively (Fig. 1). Their expression is co-regulated by the transcriptional regulator Ria1, also encoded in this cluster, which is considered as an itaconate cluster specific/narrow domain transcription factor, triggering the transcription of the itaconate biosynthesis genes [17]. Overexpression of *UMAG*_*ria1* upregulated the expression of biosynthesis core-cluster genes and transporters [17]. Additionally, the (*S*)-2-hydroxyparaconate biosynthesis gene *UMAG*_*cyp3* encoding the cytochrome P450 family 3 monooxygenase Cyp3 and *UMAG*_*rdo1* encoding a putative ring cleaving dioxygenase are adjacent to the itaconate gene cluster of *U. maydis*, the former of which converts itaconate to (*S*)-2-hydroxyparaconate [18]. Further, it was reported by Guevarra and Tabuchi that (*S*)-2-hydroxyparaconate is converted to itatartarate by a lactonase [11, 19]. *UMAG*_c*yp3* and *UMAG*_*rdo1* are not part of the core cluster and not directly upregulated by overexpression of *UMAG*_*ria1* [17]. However, all itaconate cluster genes including the two adjacent to the core cluster, *UMAG*_*cyp3* and *UMAG*_*rdo1*, are strongly upregulated during teliospore formation in the late biotrophic growth stage during plant colonization [20–22].

The number of so far undiscovered secondary metabolites produced by enzymes encoded by cryptic or orphan gene clusters are innumerably high [23, 24]. However, the availability of numerous whole fungal genome sequences and in silico gene prediction by bioinformatic algorithms, such as SMURF [25], MiBiG [26], antiSMASH [27], and FungiFun [28], allow the identification of these cryptic gene clusters. These bioinformatic tools enable ‘genome mining’ via comparison of protein sequence and structure homology. Traditional ways of activating the expression of secondary metabolites clusters include the variation in the cultivation conditions, such as medium, pH, temperature, aeration, or light, or co-cultivation with other microbes to simulate the natural expression conditions [8]. Often these physiological or ecological triggers are not sufficient to activate these clusters, and therefore several strategies have been developed to induce undiscovered silent secondary metabolite cluster [8]. The most prominent strategies are genetic engineering approaches: the overexpression of a cluster-specific transcription factor gene allowing the increased expression of the whole cluster [24]. In this case, the overexpression of the enzymes encoded within the cluster leads to diverse products, a potential challenge for natural product production [18, 24]. In addition, the endogenous promoters of secondary metabolism biosynthesis genes can be exchanged for strong inducible or constitutive promoters or global regulators can be overexpressed or deleted. A prominent example of the activation of a silent gene cluster is the overexpression of the transcriptional regulator gene *apdR* in *Aspergillus nidulans*, which induced the expression of all cluster genes, leading to the discovery of the cytotoxic aspyridones [24].

Itaconate production is naturally induced by nitrogen limitation in *U. maydis* and was also observed in other related Ustilaginaceae species such as *U. cynodontis*, *U. xerochloae*, *U. vetiveriae* that show high potential to be promising and effective itaconate producers [10, 12, 29]. However, the investigated species and strains varied in their product spectra and the amount of secreted product. Among the species, individual strains of *U. maydis* differed highly in their itaconate and (*S*)-2-hydroxyparaconate production [10]. Some of the species investigated, for example *U. vetiveriae* strain CBS 131474, produced itaconate or (*S*)-2-hydroxyparaconate only with glycerol as carbon source. Also, itaconate production varied depending on extracellular pH. While in wild type *U. maydis* itaconate production is only possible in the pH-range of 5–7, *U. cynodontis* strains also produce itaconate at pH values below 3. The genes responsible for itaconate biosynthesis and how they are regulated to explain this variation in production levels and environmental inputs are not known for these specific strains.

In the current study, 13 itaconate producers of the Ustilaginaceae family were further characterized towards their itaconate cluster sequence-function relationship. The itaconate gene clusters of these strains were identified by genome sequencing [29] and comparison to the annotated itaconate cluster of *U. maydis* strain MB215. To explore the evolutionary conservation of regulation of the itaconate cluster in respect to itaconate production by members of the Ustilaginaceae family, the phylogenetic relationship and inter-species transferability of the itaconate cluster transcription factor Ria1 was investigated. Itaconate production could be activated or enhanced by overexpression of Ria1 originating from related species. This is the first time that activation of silent itaconate clusters by overexpression of a cluster-specific transcription factor in Ustilaginaceae species other than *U. maydis* is shown.

## Results and discussion

### Variation in itaconate and (S)-2-hydroxyparaconate production among Ustilaginaceae

**Fig. 2:**
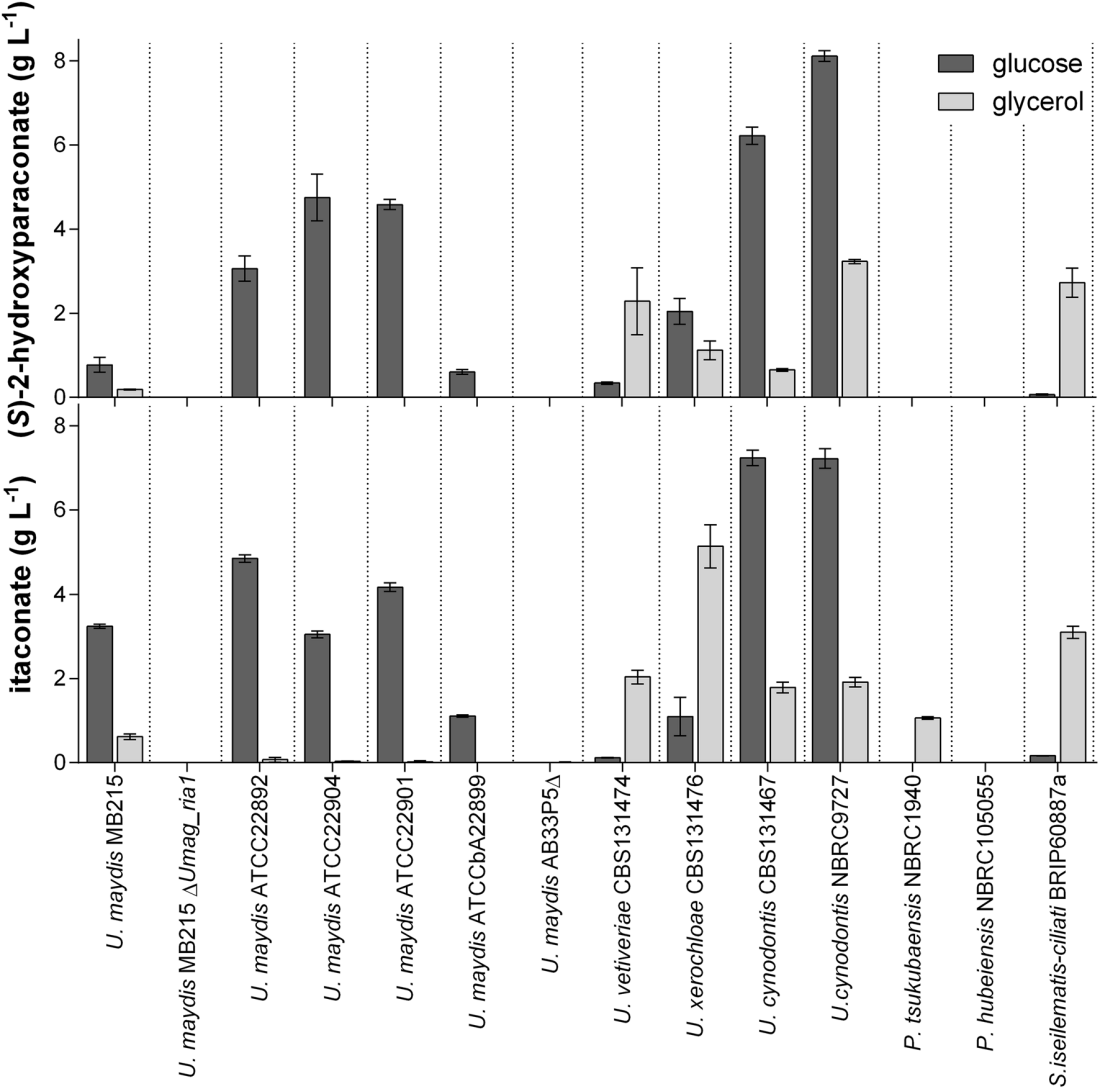
Itaconate and (*S*)-2-hydroxyparaconate production by various species in the Ustilaginaceae cultivated on glucose and glycerol. Itaconate and (*S*)-2-hydroxyparaconate concentrations after 120 h or 384 h System Duetz^®^ cultivations in screening medium with glucose or glycerol, respectively. The *U.* *maydis* Δ*Umag_ria1* mutant derived from wild type strain MB215 was used as a negative control. Error bars indicate standard deviation from the mean (n = 3)

Previous studies showed a high variation in natural itaconate production among related Ustilaginaceae species cultured on glucose and glycerol as carbon sources [10, 12]. Besides their varying amounts of product and product spectrum, they also differed in their efficiency of carbon utilization. Some of the species produced itaconate only on a single carbon source like glycerol or glucose. These differences motivated us to investigate itaconate and derivates production on glucose and glycerol in more detail (Fig. 2, Additional file 1: Fig. S1). *U.* *maydis* Δ*Umag_ria1* was used as a negative control, since the transcriptional regulator gene *ria1* is deleted and therefore itaconate production abolished. In *U.* *maydis* strain AB33P5Δ five extracellular proteases are deleted [30]. With these deletions, the strain is well suited for the secretion of heterologous or intrinsic extracellular biomass degrading CAZymes. This strain would be an optimal candidate for the synthesis of itaconate or other valuable chemicals directly from biomass-derived substrates [31]. However, it does not produce itaconate and the lack of extracellular proteases significantly reduces the growth rate of this mutant.

All wild type strains consumed at least 50% of the glucose in the 120 h except of *U.* *maydis* AB33P5Δ, which utilized 40% (Additional file 2: Fig. S2). The growth on glycerol is slower in comparison to glucose, therefore samples were taken after 384 h. At this time point, all strains consumed at least 30% of the glycerol, except of *U. maydis* AB33P5Δ, which used 13% (Additional file 2: Fig. S2). Most *U. maydis* strains produced itaconate only on glucose as the carbon source, whereas *U. vetiveriae, P. tsukubaensis,* and *S. iseilematis*-*ciliati* did so only on glycerol. *U. cynodontis* and *U. xerochloae* produced itaconate on both carbon sources. *U. maydis* AB33P5Δ, *U.* *maydis* Δ*Umag_ria1*, and *P.* *hubeiensis* did not produce itaconate at all. Since (*S*)-2-hydroxyparaconate and itatartarate are derivatives from itaconate [11], the production of these compounds was also investigated. (*S*)-2-hydroxyparaconate production of the tested strains on glucose and glycerol was similar to itaconate production (Fig. 2), with the exception of *P.* *tsukubaensis*, which only produced itaconate. Also, estimated itatartarate production levels showed a similar trend compared to (*S*)-2-hydroxyparaconate production except for *S. iseilematis*-*ciliati*, which did not to produce itatartarate (Additional file 1: Fig. S1). Previous studies showed a negative correlation between itaconate and malate production [12], therefore malate production was also determined. All strains produced malate on glucose and glycerol except for *U. maydis* AB33P5Δ and *S. iseilematis*-*ciliati*, which produced malate only on glycerol (Additional file 1: Fig. S1). In general *U.* *maydis* strains showed the highest malate titers. These results are in accordance with our previous study [10].

A possible reason for these varying titers of itaconate and its derivatives could be differences in the sequences of the itaconate and (*S*)-2-hydroxyparaconate biosynthesis genes, or the genetic inventory of these genes. Furthermore, different regulation or relative expression levels of the biosynthesis genes could cause varying production [1, 8]. Due to the targeted disruption of the genes encoding its five proteases, *U. maydis* strain AB33P5Δ is a slow growing strain in comparison to wild type and other Ustilaginaceae strains, possibly caused by different timing of the strains concerning C- or N-source utilization or their growth rate [32]. To gain a deeper understanding of the sequence-function relationship between itaconate/(*S*)-2-hydroxyparaconate biosynthesis genes and production, the genomes of 13 Ustilaginaceae were analyzed and genes related to synthesis of these secondary metabolites annotated and characterized.

### Genetic differences in the itaconate biosynthesis cluster

**Fig. 3:**
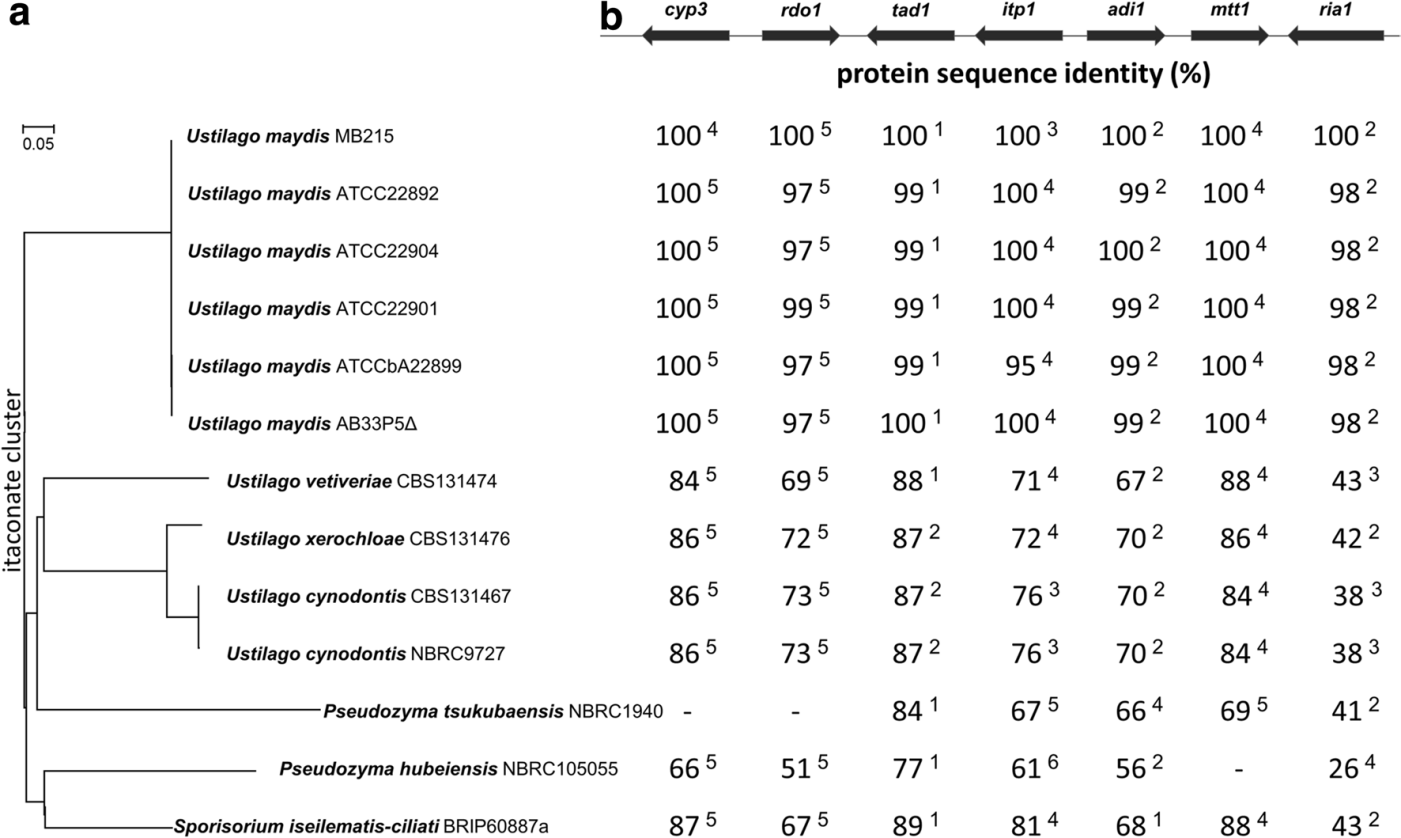
Itaconate cluster composition of selected Ustilaginaceae and a phylogenetic tree of these genes. **a** Phylogenetic tree based on the DNA sequences of itaconate clusters of different Ustilaginaceae. The optimal tree with the sum of branch length = 1.88603985 is shown. The evolutionary distances are in the units of 0.05 base substitutions per site. **b** Itaconate cluster comparison of selected Ustilaginaceae. Numbers given show sequence identity as percentage compared to the reference strain *U. maydis* MB215 using global protein sequence multiple alignment tool (BLOSUM 62). Superscript number indicate number of exons for each gene. Absent genes are indicated with a dash (-)

The Whole Genome Shotgun sequences of *Ustilago maydis* MB215 (DSM17144), *Ustilago maydis* ATCC 22892, *Ustilago maydis* ATCC22904, *Ustilago maydis* ATCC22901, *Ustilago maydis* ATCCbA22899, *Ustilago maydis* AB33P5Δ, *Ustilago vetiveriae* CBS131474, *Ustilago xerochloae* CBS131476, *Ustilago cynodontis* CBS131467, *Ustilago cynodontis* NBRC9727, *Pseudozyma tsukubaensis* NBRC1940, *Pseudozyma hubeiensis* NBRC105055, and *Sporisorium iseilematis*-*ciliati* BRIP60887a have been deposited in DDBJ/ENA/GenBank [29]. Their accession numbers are listed in “Methods”. To find the genes responsible for itaconate and (*S*)-2-hydroxyparaconate biosynthesis in these sequenced strains, the protein sequences encoded in the *U. maydis* MB215 itaconate biosynthesis cluster (GenBank KT852988.1) were used as queries against the Whole Genome Shotgun sequences database using the tBLASTn algorithm [18, 33]. Multiple hits with neighboring genes were defined as putative itaconate clusters. For cluster annotation, the highest resulting homologous sequences were further analyzed using the online tool “Augustus gene prediction” to identify start/stop codons and exons [34], followed by manual curation. Furthermore, protein sequences of *U. maydis* MB215 were compared to the predicted proteins of the investigated Ustilaginaceae using the global protein sequence multiple alignment tool (BLOSUM 62) [35] in Clone Manager 9 Professional Edition. The protein sequence identity of the investigated Ustilaginaceae proteins compared to the itaconate cluster of reference strain *U. maydis* MB215 is presented in Fig. 3b. Additionally, the phylogenetic tree based on the DNA sequences of itaconate clusters of different Ustilaginaceae indicates the phylogenetic relationship among the chosen strains (Fig. 3a).

Exact phylogenetic classification among Ustilaginaceae is challenging, with several species being renamed based on new analysis of indicator genes such as nuclear ribosomal RNA genes [39–41]. Wang et al. especially mentioned that strains in the genus *Pseudozyma* have an uncertain phylogenetic position due to the taxonomic confusion between their teleomorphic genera [39]. Therefore, a phylogenetic relation is shown based on the DNA sequence of the itaconate cluster (Fig. 3a).

In all sequenced organisms except *P.* *tsukubaensis* and *P.* *hubeiensis*, the complete gene cluster for itaconate synthesis and conserved synteny of all genes (gene orientation and chromosome) were identified (Fig. 3b). The cluster in *P.* *tsukubaensis* does not contain *rdo1* and *cyp3*. For these two genes no likely homologous candidate was found elsewhere in genome, explaining the lack of (*S*)-2-hydroxyparaconate and itatartarate production in this strain (Fig. 2). In *P.* *hubeiensis*, *mtt1* is not present in the itaconate cluster or its direct surrounding DNA regions. In *U.* *maydis* MB215, deletion of *UMAG*_*mtt1* led to a strong decrease in itaconate production [17]. This transporter, which putatively shuttles malate and *cis*-aconitate between the mitochondria and the cytoplasm, is the rate-limiting step in itaconate biosynthesis in *U. maydis* MB215 [42]. Since itaconate formation was not completely abolished by *Umag*_*mtt1* deletion in *U.* *maydis*, most likely other less specialized, and therefore less efficient, transport proteins substituted its function, as most eukaryotic mitochondrial transporters have a diverse substrate spectrum with different affinities [43]. At least one similar mitochondria tricarboxylate transporter gene is present in the genome of *P. hubeiensis*, which could take over the function of Mtt1. This gene showed 54% sequence similarity on protein level in comparison to *Umag*_*mtt1* and 98% to *Umag_02365* upon tBLASTn analysis [33]. The latter gene, *Umag_02365*, is known to be one of two related mitochondrial citrate transporters in *U. maydis*, with redundant function to *Umag*_*mtt1* [42]. This may explain why *P. hubeiensis* failed to produce itaconate.

**Fig. 4:**
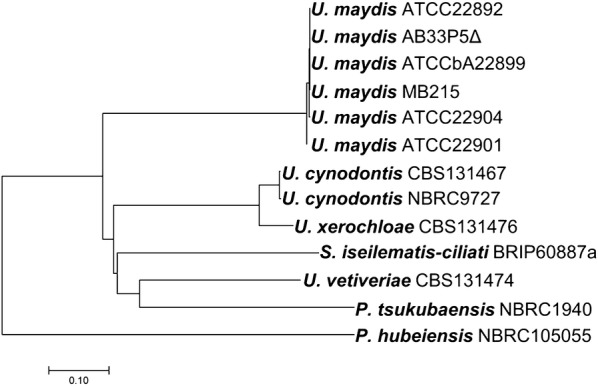
Phylogenetic tree of the Ria1 transcriptional regulator of different Ustilaginaceae based on similarities and differences in their protein sequence. The optimal tree with the sum of branch length = 2.49320505 is shown. The tree is drawn to scale, with branch lengths in the same units as those of the evolutionary distances used to infer the phylogenetic tree. The evolutionary distances are in the units of 0.1 amino acid substitutions per site

In general, the conservation of a protein sequence could point to its evolutionary origin. The comparison showed that among the tested *U. maydis* strains the itaconate cluster is conserved. At the DNA level the clusters in different *U.* *maydis* strains are > 98% similar and the clusters of the two *U. cynodontis* strains have 99% sequence identity on DNA level. For the other species, the sequence identity of proteins encoded by the itaconate and (*S*)-2-hydroxyparaconate biosynthesis (*cyp3*, *tad1* and *adi1*) and transporters (*itp1* and *mtt1*) genes were mostly conserved in a range of 56–89% compared to the *U. maydis* MB215 sequence. The most divergent protein of the itaconate cluster is Ria1, a transcription factor of approximately 380 amino acids. The annotated *Uc_ria1* of both *U.* *cynodontis* strains encode a transcription factor of 471 amino acids. A conserved helix-loop-helix structural motif could be found in all 13 regulators approximately in position 100-AA by SMART analysis, which is a characteristic DNA-binding motif for one of the largest families of dimerizing transcription factors [44, 45]. The phylogenetic tree of the predicted Ria1 transcriptional regulators is shown in Fig. 4. Ria1 proteins of *U. maydis* species are very closely related. *U.* *cynodontis* and *U.* *xerochloae* are closely related [46], which is reflected in the relatedness of their Ria1 proteins. However, *U.* *maydis* and *U.* *vetiveriae* are phylogenetically closely related as well [46], even though their Ria1 proteins are only 43% identical. This may indicate that the amino acid sequence of Ria1 proteins is evolving faster than its actual function, for which just the DNA-binding motif is essential. The Ria1 sequence of *P.* *hubeiensis* is phylogenetically the most distant of the species compared. In general, no accurate subcategorization of the transcription factor according the species is possible. A reason might be the aforementioned difficulties in categorization of the Ustilaginaceae.

In summary, all tested strains have the genetic inventory for itaconate biosynthesis, and the synteny of the itaconate cluster is preserved in most of the investigated Ustilaginaceae. *P. tsukubaensis* and *P.* *hubeiensis* do not possess the complete itaconate cluster, partly explaining the differences in product spectrum. However, the variable itaconate and (*S*)-2-hydroxyparaconate titer, especially among different *U.* *maydis* strains with highly similar clusters, could not be explained. As different regulation or expression levels might be responsible for these production differences, the Ria1 transcriptional regulator of the tested Ustilaginaceae were investigated in more detail.

### Inter-species transferability of Ria1 regulator

The itaconate clusters of the tested strains are mostly conserved, while production levels of itaconate differ. As one example, the *U.* *maydis* AB33P5Δ gene cluster is 98% similar at the DNA level to that of *U. maydis* ATCCbA22899; however, strain AB33P5Δ does not produce itaconate or (*S*)-2-hydroxyparaconate while strain ATCCbA22899 does. Probably in some strains, like *U.* *maydis* AB33P5Δ, *ria1* is functional but not expressed. To test whether production differences are a result of different regulation, the inter-species transferability of Ria1 was investigated by overexpression of various *ria1* genes to activate the production of itaconate. We chose the itaconate cluster regulator genes *Umag_ria1*, *Uc_ria1*, *Pt_ria1*, and *Si_ria1* of *U. maydis* MB215, *U. cynodontis* NBRC9727, *P. tsukubaensis*, and *S.* *iseilematis*-*ciliati*, respectively, due to their considerable differences in the sequences of both the itaconate cluster and Ria1. These regulators were expressed under control of the constitutive promoter P_*etef*_ in *U.* *maydis* MB215, *U.* *maydis* AB33P5Δ, *U.* *vetiveriae*, *U.* *xerochloae*, *U.* *cynodontis* CBS131467, *U.* *cynodontis* NBRC9727, *P.* *tsukubaensis*, *P.* *hubeiensis*, and *S.* *iseilematis*-*ciliati*, as well as in the control strain *U. maydis* MB215 Δ*Umag_ria1*. Successful integration was verified by PCR.

**Fig. 5:**
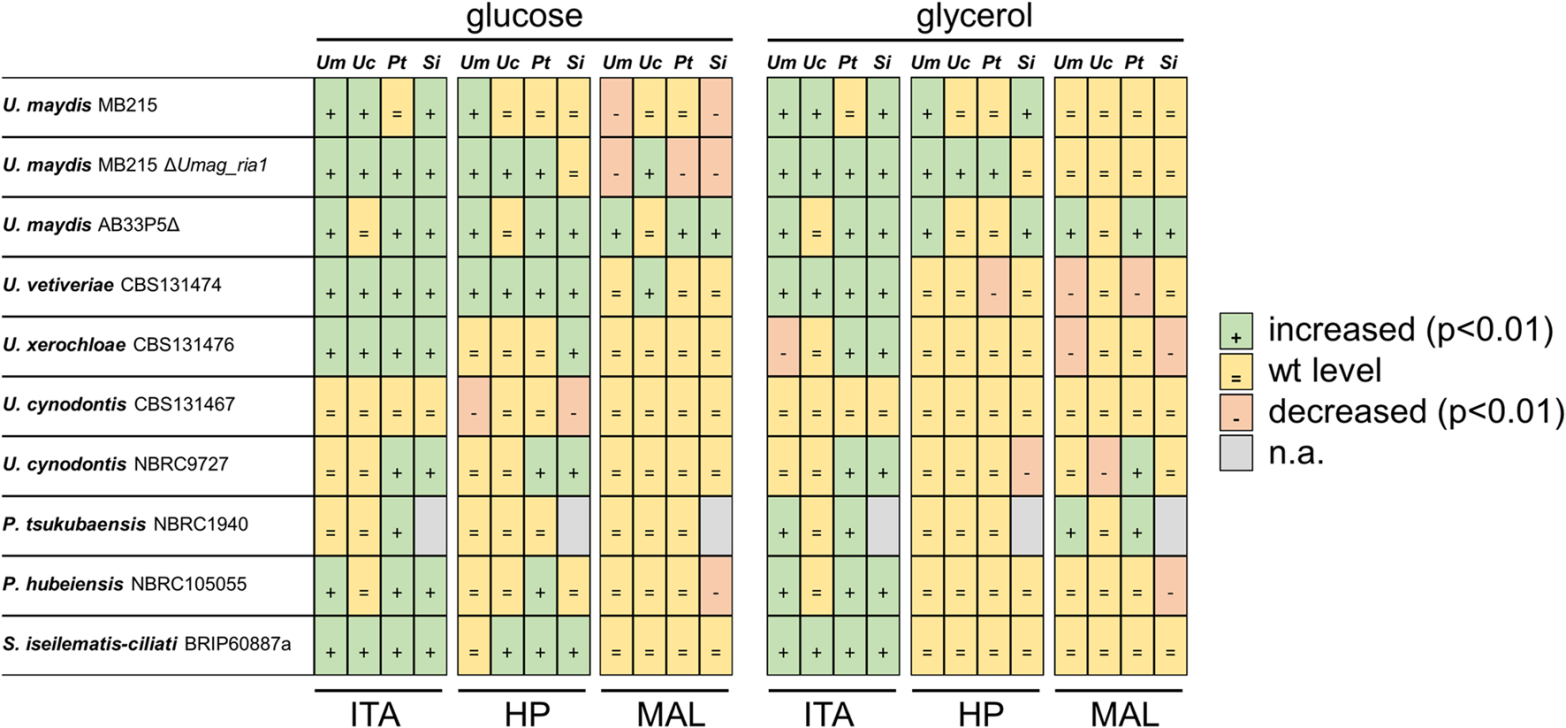
Overview of the influence of overexpression of *Umag_ria1*, *Uc_ria1*, *Pt_ria1* and *Si_ria1*, on itaconate (ITA), (*S*)-2-hydroxyparaconate (HP), and malate (MAL) production. Differences in production were determined after 120 h or 384 h System Duetz^®^ cultivations in screening medium containing either glucose or glycerol as the sole carbon source

**Fig. 6:**
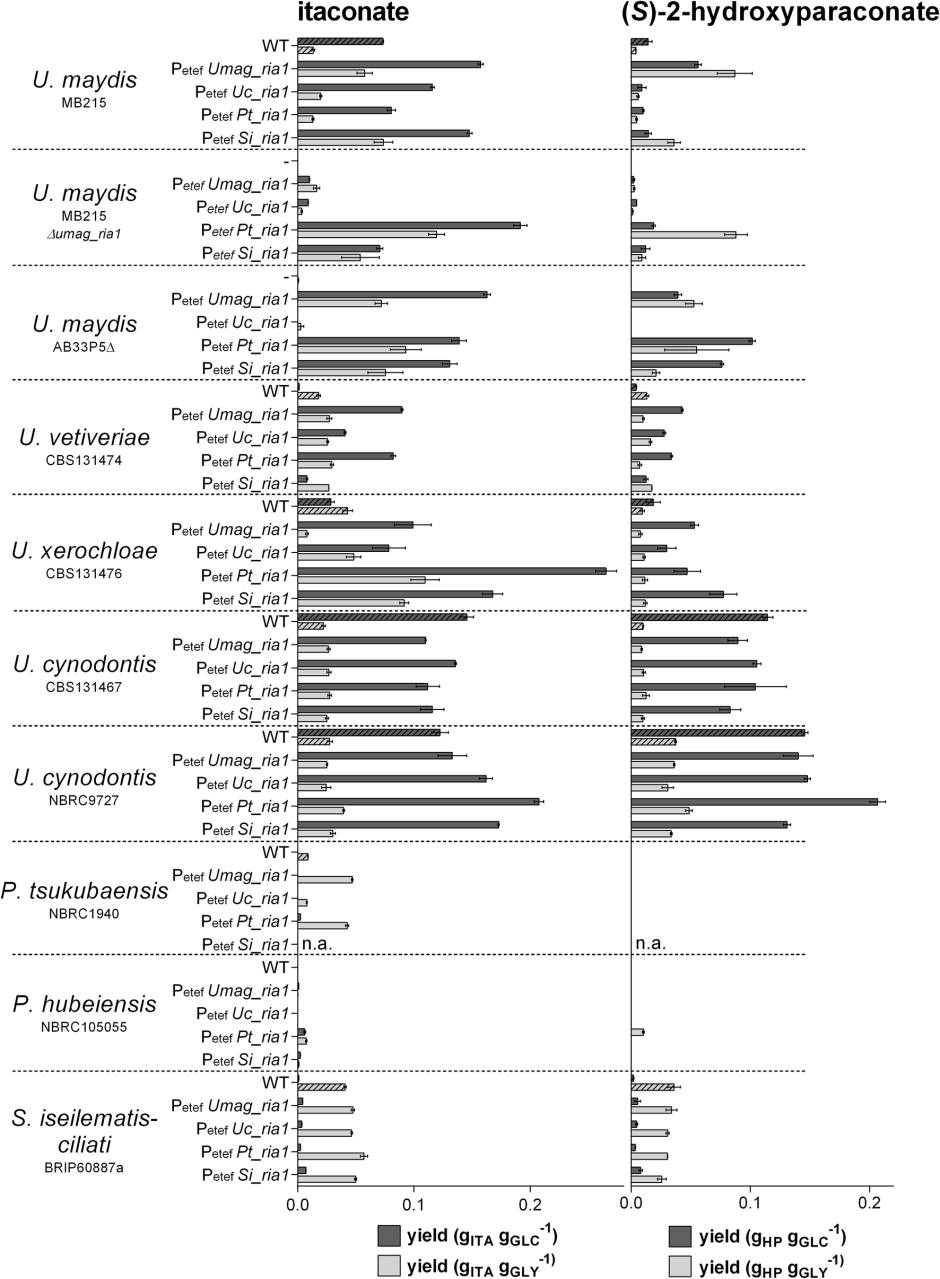
Itaconate and (*S*)-2-hydroxyparaconate production by various *Ustilaginaceae* species and their mutants transformed with *Umag_ria1*, *Uc_ria1*, *Pt_ria1*, *Si_ria1*. Itaconate (g_ITA_ g_GLC_^−1^, g_ITA_ g_GLY_^−1^) and (*S*)-2-hydroxyparaconate (g_HP_ g_GLC_^−1^ g_HP_ g_GLY_^−1^) yield after 120 h or 384 h System Duetz^®^ cultivations in screening medium containing glucose (GLC) and glycerol (GLY), respectively. A dash (–) indicates the negative control without an overexpression construct. Error bars indicate standard deviation from the mean (n = 3)

All strains tested consumed at least 35% of the applied glucose after 120 h and 30% of the applied glycerol after 384 h except of *U. maydis* AB33P5Δ, which used 13% glycerol (Additional file 2: Fig. S2). A summary of the activation experiments is shown in Fig. 5 and Additional file 3: Fig. S3. The itaconate and (*S*)-2-hydroxyparaconate production yield (gram product per gram substrate) of the activated strains was determined on both glucose and glycerol (Fig. 6) as well as the malate yield and the estimated relative itatartarate production (Additional file 4: Fig. S4). In *U. maydis* MB215 Δ*Umag_ria1*, itaconate production could be restored by expression of all tested regulators (*Umag_ria1*, *Uc_ria1*, *Pt_ria1*, and *Si_ria1*), demonstrating the functionality of this expression system, as well as their transferability of the genes between related species. It should be noted that quantitative differences in production level may be caused by different copy number, or by the random ectopic integration locus, of the integrated regulator, which were not determined in detail. Thus, these results should be viewed mostly in a qualitative manner. In strains that do not produce itaconate on glucose, such as *U.* *maydis* AB33P5Δ (derivative of *U. maydis* FB1), *U.* *vetiveriae*, *P. tsukubaensis*, *P.* *hubeiensis*, and *S.* *iseilematis*-*ciliati* itaconate production could be activated by expression of all tested regulators, except *Uc_ria1*. This suggests that in these wild type strains the itaconate cluster genes are silent, because the regulator gene *ria1* is silent and not transcribed. Constitutive expression of the itaconate regulator *ria1*, even originating from different species, activated the expression of the itaconate cluster genes, which resulted in itaconate production.

As already encountered for the wild type strains, (*S*)-2-hydroxyparaconate and itatartarate production correlated with itaconate production. In general, activation or enhancement of itaconate biosynthesis also activated or enhanced (*S*)-2-hydroxyparaconate and itatartarate biosynthesis (Fig. 5, Additional files 3 and 4: Fig. S3 and Fig. S4). An exception is *P. tsukubaensis* that does not possess the (*S*)-2-hydroxyparaconate biosynthesis genes *rdo1* and *cyp3*, and therefore (*S*)-2-hydroxyparaconate and itatartarate are not produced in the activated strains. Zambanini et al. showed a negative correlation of itaconate and malate biosynthesis after overexpression of *Umag_ria1* in *U. vetiveriae* CBS131474 on glycerol [12]. This is in line with our results. However, for the other tested Ustilaginaceae this negative correlation could not be shown, as in most activated strains malate production resembled the wild type level (Fig. 5, Additional file 4: Fig. S4).

Comparing the successfully activated or improved strains, those expressing *Uc_ria1* perform considerably less well than strains expressing the other regulators. Deletion of *Uc_ria1* in *U.* *cynodontis* NBRC9727 completely abolished itaconate production (data not shown), indicating that *Uc_ria1* is essential for itaconate production. However, *U.* *cynodontis* NBRC9727 Δ*Uc_ria1* could not be complemented by *Uc_ria1* under control of the constitutive promoter P_*etef*_, although complementation experiments under control of the native promotor P_*Uc_ria1*_ and terminator T_*Uc_ria1*_ and a random genome integration was successfully (data not shown). This indicates that the integration locus of genes under control of P_*etef*_ plays a crucial role for heterologous expression in *U.* *cynodontis* and that the chosen expression cassette design may have affected the outcome of *ria1* overexpression in different hosts. In *U.* *cynodontis* strains itaconate production could not be considerably enhanced, even by overexpression of the native regulator *Uc_ria1*. In contrast, strains more closely related phylogenetically with a lower wild type production level, such as *U.* *xerochloae*, could still enhance itaconate production by overexpression of *Uc_ria1*. Since the *U.* *cynodontis* strains were the best performing wild types, it might be possible that the natural expression level of the cluster genes is already at a high level, and the rate limiting factor lies upstream of the itaconate production pathway. Alternatively, induction by Ria1 is in *U. cynodontis* already at its maximum.

Although the itaconate production of *P.* *tsukubaensis* and *S.* *iseilematis*-*ciliati* strains is comparatively low, the regulators *Pt_ria1* and *Si_ria1* seem to be the most universally applicable, since they improved itaconate production in 80% of the tested strains when cultured on glucose or glycerol. Therefore, *Pt_ria1* and *Si_ria1* might open new possibilities to activate itaconate production in other species through heterologous gene expression approaches.

In general, the differences in itaconate production in strains expressing the same regulator could have several explanations. The chosen constitutive promoter P_*etef*_ is a modified *tef* promoter controlling transcription of the gene for the translation elongation factor 2 of *U.* *maydis* [47]. It may be less efficient in other Ustilaginaceae than in *U.* *maydis*. However, its functionality was verified in *U.* *trichophora* [48] and *U.* *vetiveriae* [12]. As mentioned before, different copy numbers of the integrated regulators can cause differences in transcription levels and therefore in production levels. Especially for results on glycerol showing an overall similar trend than on glucose, different growth kinetics, including, growth rates, and substrate uptake rates can cause differences in itaconate production. The growth rate on glycerol of Ustilaginaceae is lower in comparison to that on glucose, hence less nitrogen for biomass synthesis per time is required, which subsequently influences nitrogen limitation during cultivation. Nitrogen limitation is necessary for natural induction of itaconate production in *U.* *maydis* [49]. The maximum theoretical yield of itaconate production is directly related to the consumed C/N ratio, and thus poor growth (low growth rate) could result in a lower yield given the chosen cultivation time. Altogether, itaconate production could be activated or enhanced by overexpression of Ria1 originating from a related species, even though the chosen Ria1 protein sequences are very dissimilar. This is the first time that activation of silent itaconate clusters by overexpression of a cluster-specific transcription factor across species and even genus boundaries was shown.

**Fig. 7:**
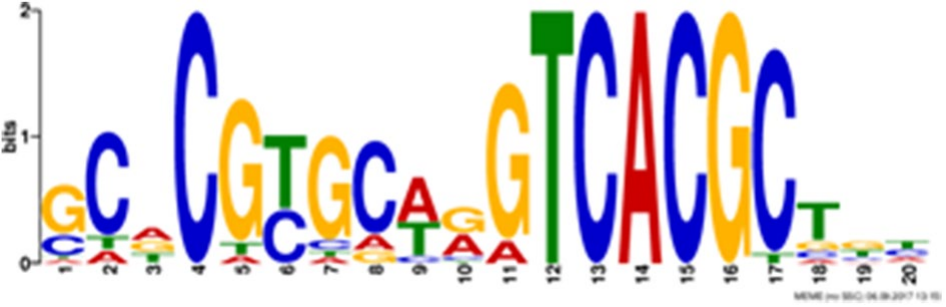
Common motif within the promoter regions of the itaconate cluster genes in all tested Ustilaginaceae was identified by MEME analysis [50]

Since overexpression of Ria1 upregulates all genes of the itaconate core cluster in *U. maydis* MB215 [17], promoter regions of the co-regulated genes will likely have a common conserved regulator binding domain. In this study the phylogenetic relatedness and the feasible inter-species transferability of Ria1 regulator originating from related species could be shown. To identify potential common regulatory sequences, in silico analysis for conserved sequence motifs was performed using the MEME algorithm Version 4.12.0 under standard settings [50]. This analysis revealed that promoters of Ria1-regulated genes share a putative conserved Ria1 binding domain with a short consensus sequence (CN[T/C]NNNN[G/A]TCACG[C/T]) (Fig. 7). This sequence can be found in all tested Ustilaginaceae in either orientation in the promoter regions of all annotated cluster genes in at least one copy with an average E-value of 1.8 × 10^−83^. Interestingly, none of the *ria1* promoters themselves contain this element. Since most of the tested regulators do not seem to be very species-specific, this site likely binds regulators from multiple species. Although the role of this motif as the binding site for Ria1 needs to be confirmed by biochemical methods, its occurrence in the sequenced wild type strains (*U. maydis* MB215 (DSM17144), *U. vetiveriae* CBS131474, *U. xerochloae* CBS131476, *U. cynodontis* CBS131467, *P. tsukubaensis* NBRC1940 and *P. hubeiensis* NBRC105055, and *S. iseilematis*-*ciliati* BRIP60887a) strongly suggests that in spite of the relatively low amino acid sequence similarity of Ria1 in these species, the function of this regulator is the same.

## Conclusion

This study indicates phenotypically that itaconate production differences among related Ustilaginaceae species are based on different transcriptional regulation of the itaconate cluster genes, governed in turn by the expression level of Ria1. All tested strains have the genetic equipment for itaconate production; also, itaconate non-producers. However, in some strains the itaconate clusters are silent, because the itaconate regulator *ria1* is silent. By overexpression of itaconate cluster-specific transcription factors Ria1 originating from related species, we could activate silent itaconate clusters, even though the amino acid sequences of Ria1 regulators are relatively dissimilar. In additional to the silent itaconate clusters being activated, itaconate production in weak producers could be enhanced up to 4-fold. Especially, the activated form of *U.* *maydis* strain AB33P5Δ might be a promising candidate for the combination of biomass degradation and itaconate production in one strain [31]. As such, this study contributes to demonstrating the industrial applicability of Ustilaginaceae for the biotechnological production of itaconate, and also suggests that activation of silent secondary metabolite clusters can be achieved in a range of related species with reduced genetic engineering efforts.

## Methods

### Strains and culture conditions

All strains used in this work are listed in Table 1.

### Ria1 overexpression constructs

To generate the overexpression construct, the backbone of the plasmid P_etef_-*ria1*-*cbx* from *Escherichia coli* Top10+* Petef Umag_ria1*-*Cbx* was amplified by PCR with the primer pair HT-212 and HT-213 (Table 1, Additional file 5: Table S1). The genes encoding the transcription factors Ria1 from *Pseudozyma tsukubaensis*, *Ustilago cynodontis* NBRC9727, and *Sporisorium iseilematis*-*ciliati* were amplified by PCR using the primer pairs HT-214/HT-215, HT-218/HT-219, and HT-216/HT-217, respectively (Additional file 5: Table S1). Gibson cloning with backbone and different *ria1* genes was conducted to obtain the plasmids P_etef_Pt_ria1, P_etef_Uc_ria1, and P_etef_Si_ria1, respectively [51]. Enzyme digestion and PCR ensured correct assembly.

**Table 1:** Strains used in this study

Strain designation	Resistance	Reference/GenBank Accession number
*Escherichia coli* Top10 + P_*etef*_ *Umag_ria1*-*Cbx*	Ampicillin	[54]
*Escherichia coli* NEB^®^ 5a P_*etef*_ *Uc_ria1*-*Cbx*	Ampicillin	This study
*Escherichia coli* NEB^®^ 5a P_*etef*_ *Pt_ria1*-*Cbx*	Ampicillin	This study
*Escherichia coli* NEB^®^ 5a P_*etef*_ *Si_ria1*-*Cbx*	Ampicillin	This study
*Ustilago maydis* DSM17144 (*Ustilago maydis* MB215)	Wild type	AACP00000000
*Ustilago maydis* DSM17144 P_etef_ *Umag_ria1*	Carboxin	This study
*Ustilago maydis* DSM17144 P_etef_ *Uc_ria1*	Carboxin	This study
*Ustilago maydis* DSM17144 P_etef_ *Pt_ria1*	Carboxin	This study
*Ustilago maydis* DSM17144 P_etef_ *Si_ria1*	Carboxin	This study
*Ustilago maydis* DSM17144 Δ*Umag_ria1*	Hygromycin	[54]
*Ustilago maydis* DSM17144 Δ*Umag_ria1* P_etef_ *Umag_ria1*	Hygromycin, carboxin	[54]
*Ustilago maydis* DSM17144 Δ*Umag_ria1* P_etef_ *Uc_ria1*	Hygromycin, carboxin	This study
*Ustilago maydis* DSM17144 Δ*Umag_ria1* P_etef_ *Pt_ria1*	Hygromycin, carboxin	This study
*Ustilago maydis* DSM17144 Δ*Umag_ria1* P_etef_ *Si_ria1*	Hygromycin, carboxin	This study
*Ustilago maydis* ATCC 22892	Wild type	LYOO00000000
*Ustilago maydis* ATCC22904	Wild type	LZQT00000000
*Ustilago maydis* ATCC22901	Wild type	LZNJ00000000
*Ustilago maydis* ATCCbA22899	Wild type	LYZD00000000
*Ustilago maydis* AB33P5Δ	Wild type	[30] /LZQU00000000
*Ustilago maydis* AB33P5Δ P_etef_ *Umag_ria1*	Carboxin	This study
*Ustilago maydis* AB33P5Δ P_etef_ *Uc_ria1*	Carboxin	This study
*Ustilago maydis* AB33P5Δ P_etef_ *Pt _ria1*	Carboxin	This study
*Ustilago maydis* AB33P5Δ P_etef_ *Si_ria1*	Carboxin	This study
*Ustilago vetiveriae* CBS131474	Wild type	MAIM00000000
*Ustilago vetiveriae* CBS131474 P_etef_ *Umag_ria1*	Carboxin	[12]
*Ustilago vetiveriae* CBS131474 P_etef_ *Uc_ria1*	Carboxin	This study
*Ustilago vetiveriae* CBS131474 P_etef_ *Pt _ria1*	Carboxin	This study
*Ustilago vetiveriae* CBS131474 P_etef_ *Si_ria1*	Carboxin	This study
*Ustilago xerochloae* CBS131476	Wild type	MAIN00000000
*Ustilago xerochloae* CBS131476 P_etef_ *Umag_ria1*	Carboxin	This study
*Ustilago xerochloae* CBS131476 P_etef_ *Uc_ria1*	Carboxin	This study
*Ustilago xerochloae* CBS131476 P_etef_ *Pt _ria1*	Carboxin	This study
*Ustilago xerochloae* CBS131476 P_etef_ *Si_ria1*	Carboxin	This study
*Ustilago cynodontis* CBS131467	Wild type	LZQV00000000
*Ustilago cynodontis* CBS131467 P_etef_ *Umag_ria1*	Carboxin	This study
*Ustilago cynodontis* CBS131467 P_etef_ *Uc_ria1*	Carboxin	This study
*Ustilago cynodontis* CBS131467 P_etef_ *Pt _ria1*	Carboxin	This study
*Ustilago cynodontis* CBS131467 P_etef_ *Si_ria1*	Carboxin	This study
*Ustilago cynodontis* NBRC9727	Wild type	LZZZ00000000
*Ustilago cynodontis* NBRC9727 P_etef_ *Umag_ria1*	Carboxin	This study
*Ustilago cynodontis* NBRC9727 P_etef_ *Uc_ria1*	Carboxin	This study
*Ustilago cynodontis* NBRC9727 P_etef_ *Pt _ria1*	Carboxin	This study
*Ustilago cynodontis* NBRC9727 P_etef_ *Si_ria1*	Carboxin	This study
*Pseudozyma tsukubaensis* *NBRC1940*	Wild type	MAIP00000000
*Pseudozyma tsukubaensis NBRC1940* P_etef_ *Umag_ria1*	Carboxin	This study
*Pseudozyma tsukubaensis* *NBRC1940* P_etef_ *Uc_ria1*	Carboxin	This study
*Pseudozyma tsukubaensis* *NBRC1940* P_etef_ *Pt _ria1*	Carboxin	This study
*Pseudozyma hubeiensis* *NBRC105055*	Wild type	MAIO00000000
*Pseudozyma hubeiensis NBRC105055* P_etef_ *Umag_ria1*	Carboxin	This study
*Pseudozyma hubeiensis* *NBRC105055* P_etef_ *Uc_ria1*	Carboxin	This study
*Pseudozyma hubeiensis* *NBRC105055* P_etef_ *Pt_ria1*	Carboxin	This study
*Pseudozyma hubeiensis* *NBRC105055* P_etef_ *Si_ria1*	Carboxin	This study
*Sporisorium iseilematis*-*ciliati* *BRIP60887a*	Wild type	MJEU00000000
*Sporisorium iseilematis*-*ciliati BRIP60887a* P_etef_ *Umag_ria1*	Carboxin	This study
*Sporisorium iseilematis*-*ciliati* *BRIP60887a* P_etef_ *Uc_ria1*	Carboxin	This study
*Sporisorium iseilematis*-*ciliati* *BRIP60887a* P_etef_ *Pt _ria1*	Carboxin	This study
*Sporisorium iseilematis*-*ciliati* *BRIP60887a* P_etef_ *Si_ria1*	Carboxin	This study

### Overexpression of Ria1

For random integration of the *ria1* overexpression constructs into the genome of the different Ustilaginaceae, the different plasmids (P_etef_ *Pt_ria1*, P_etef_ *Si_ria1*, P_etef_ *Uc_ria1* and P_etef_ *Umag_ria1*-cbx) were linearized by SspI except of P_etef_ *Pt_ria1*, which was linearized by BsrgI. Integration of the linearized overexpression construct in the different Ustilaginaceae was conducted by protoplasts transformation according to Tsukuda et al. [52]. To confirm plasmid integration, the *ria1* was amplified by PCR using the primer Potef-fwd and Tnos-rev.

Shaking cultures were performed in the System Duetz^®^ (24 well plates) with a filling volume of 1.5 mL (d = 50 mm, n = 300 rpm, T = 30 °C and Φ = 80%) [53]. The screening medium contained 50 g L^−1^ glucose or 100 g L^−1^ glycerol, 0.8 g L^−1^ NH_4_Cl, 0.2 g L^−1^ MgSO_4_·7H_2_O, 0.01 g L^−1^ FeSO_4_·7H_2_O, 0.5 g L^−1^ KH_2_PO_4_, 1 mL L^−1^ vitamin solution, 1 mL L^−1^ trace element solution, and as buffer 132 g L^−1^ calcium carbonate [10]. Cultures were parallelly inoculated into multiple plates and for each sample point a complete plate was taken as sacrificial sample in order to ensure continuous oxygenation.

### Analytical methods

Cell densities were measured by determining the absorption at 600 nm with a Unico spectrophotometer 1201.

Products and substrates in the supernatants were analyzed in a DIONEX UltiMate 3000 High Performance Liquid Chromatography System (Thermo Scientific, Germany) with an ISERA Metab AAC column 300 × 7.8 mm column (ISERA, Germany). As solvent 5 mM H_2_SO_4_ with a flow rate of 0.6 mL min^−1^ and a temperature of 40 °C was used. All samples were filtered with Acrodisc^®^ Syringe Filters (GHP, 0.20 µm, Ø 13 mm). Itaconate, (*S*)-2-hydroxyparaconate, malate, and itatartarate were determined with a DIONEX UltiMate 3000 Variable Wavelength Detector set to 210 nm, glycerol and glucose with a refractive index detector SHODEX RI-101 (Showa Denko Europe GmbH, Germany). Itaconate, malate, (*S*)-2-hydroxyparaconate, glucose, and glycerol were identified via retention time and UV/RI quotient compared to corresponding standards. Synthesized (*S*)-2-hydroxyparaconate (purity ~ 70%) was used as the HPLC standard for quantification and hence the indicated (*S*)-2-hydroxyparaconate values should be taken as rough estimates only [18]. Since no standards of itatartarate are commercially available this compound was analyzed relatively based on HPLC peak area (mAU*min) using the UV detector. All values are the arithmetic mean of at three biological replicates. Error bars indicate the standard deviation from the mean. Statistical analysis was performed using unequal variances *t* test with unilateral distribution (*P* values < 0.01 were considered significant and indicated in figures with *).

### Genome sequencing

Genomic DNA was isolated by standard phenol–chloroform extraction [55]. Eurofins Genomics (Ebersberg, Germany) created the library using the NEBNext^®^ Ultra DNA Library Prep Kit for Illumina^®^ (Art No E7370), and sequenced the library using an llumina HiSeq 2500 machine with TruSeq SBS kit v3 both according to manufacturer’s instructions. The sequencing mode was 1x100 and the processing used the HiSeq Control software 2.0.12.0 RTA 1.17.21.3 bcl2fastq-1.8.4. Quality check of the sequence data was performed with FastQC (Version 0.11.2). The SPAdes-3.7.0-Linux pipeline was used for de novo genome assembly of single-read libraries and read error or mismatch correction including BayesHammer, IOnHammer, SPAdes, MismatchCorrector, dipSPAdes, and truSPAdes. The k-mer size was determined to 55 using VelvetOptimiser Version 2.2.5. The Whole Genome Shotgun sequences have been deposited in DDBJ/ENA/GenBank. Their accession numbers are listed in Table 1.

### Phylogenetic analyses

The evolutionary history of itaconate cluster DNA sequences was inferred using the Neighbor-Joining method [36] after alignment via ClustalW algorithm with the MEGA 7: Molecular Evolutionary Genetics Analysis version 7.0 for bigger datasets Alignment Explorer. The optimal tree with the sum of branch length = 1.88603985 is shown. The tree is drawn to scale, with branch lengths in the same units as those of the evolutionary distances used to infer the phylogenetic tree. The evolutionary distances were computed using the Maximum Composite Likelihood method [37] and are in the units of the number of base substitutions per site. The analysis involved 13 nucleotide sequences. All positions containing gaps and missing data were eliminated. There were a total of 10874 positions in the final dataset. Evolutionary analyses were conducted in MEGA7 [38].

For the phylogenetic tree of Ria1, protein sequences were aligned via ClustalW (codon) algorithm with MEGA 7 [38]. The evolutionary history was inferred using the Neighbor-Joining method [36]. The optimal tree with the sum of branch length = 2.49320505 is shown. The tree is drawn to scale, with branch lengths in the same units as those of the evolutionary distances used to infer the phylogenetic tree. The evolutionary distances were computed using the Poisson correction method [56] and are in the units of the number of amino acid substitutions per site. The analysis involved 13 amino acid sequences. All positions containing gaps and missing data were eliminated. There were a total of 269 positions in the final dataset. Evolutionary analyses were conducted in MEGA7 [38].

## Additional files

**Additional file 1: Fig. S1.** Malate and itatartarate production by various Ustilaginaceae on glucose and glycerol. Malate concentrations and itatartarate UV area after 120 h or 384 h System Duetz^®^ cultivations in screening medium with glucose or glycerol, respectively. *U. maydis* MB215 Δ*Umag_ria1* was used as a negative control. Error bars indicate standard deviation from the mean (n = 3).

**Additional file 2: Fig. S2.** Glucose and glycerol consumption by various Ustilaginaceae. Glucose and glycerol consumption in % after 120 h or 384 h System Duetz^®^ cultivations in screening medium with glucose or glycerol, respectively. Error bars indicate standard deviation from the mean (n = 3).

**Additional file 3: Fig. S3.** Schematic overview of the influence of overexpression of *Umag_ria1*, *Uc_ria1*, *Pt_ria1* and *Si_ria1*, on itatartarate (**ITT**) production. Differences in itatartarate production were determined after 120 h or 384 h System Duetz^®^ cultivations in screening medium containing glucose or glycerol, respectively.

**Additional file 4: Fig. S4.** Malate and itatartarate production by various *Ustilaginaceae* and their mutants transformed with *Umag_ria1*, *Uc_ria1*, *Pt_ria1*, *Si_ria1*. Malate (g_Mal_ g_GLC_^−1^, g_ITA_ g_GLY_^−1^) yield and itatartarate titer after 120 h or 384 h System Duetz^®^ cultivations in screening medium containing glucose (GLC) and glycerol (GLY), respectively. A dash (–) indicates the negative control without an overexpression construct. Error bars indicate standard deviation from the mean (n = 3).

**Additional file 5: Table S1.** Oligonucleotides used for overexpression constructs.
