# Supplementary figures and images for: Evolutionary freedom in the regulation of the conserved itaconate cluster by Ria1 in related Ustilaginaceae
Source: Fungal Biol Biotechnol. 2018 Jul 28;5:14. doi: 10.1186/s40694-018-0058-1 (PMC6064134; doi:10.1186/s40694-018-0058-1)

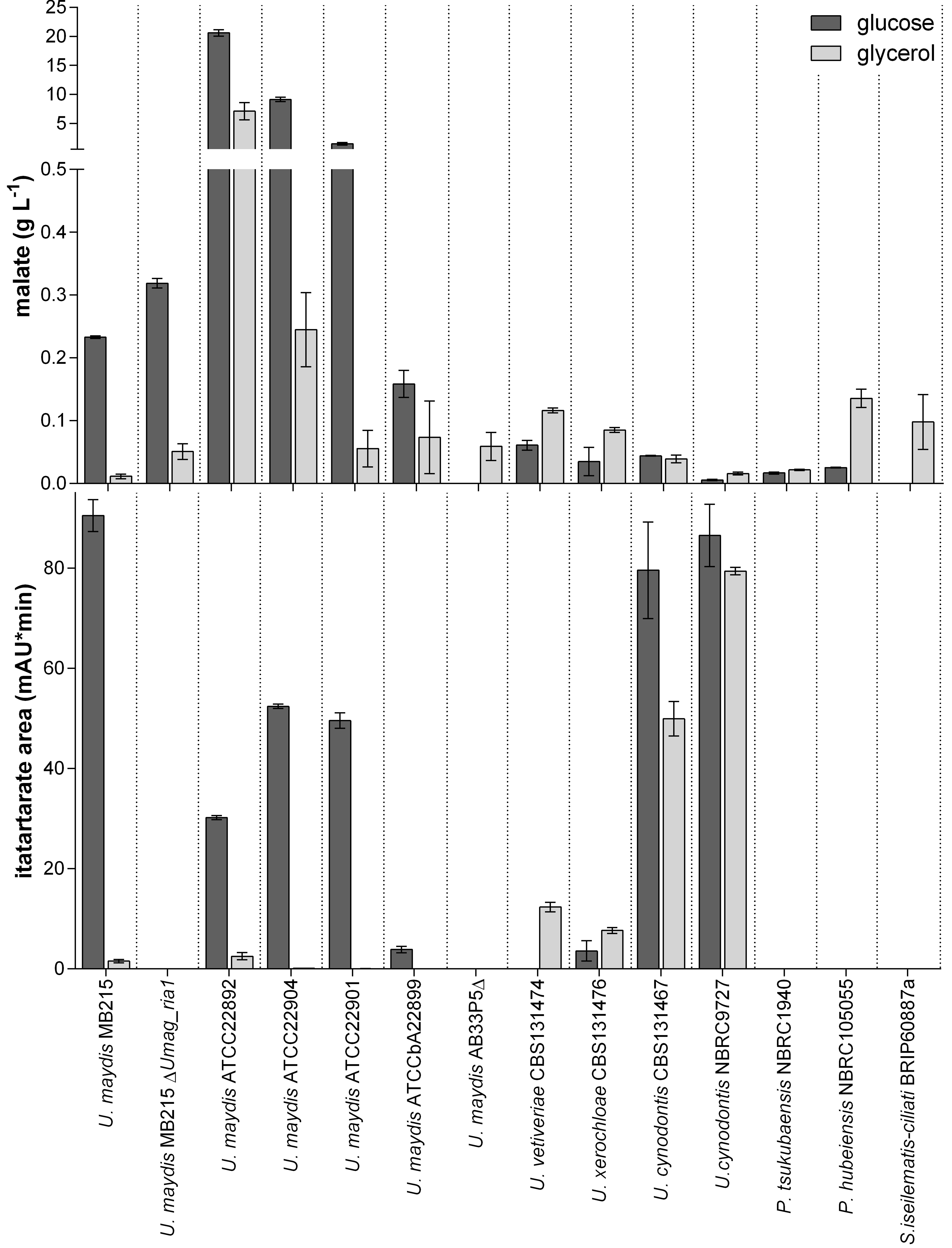

Supplement: Supplementary file 1 — Additional file 1: Fig. S1. Malate and itatartarate production by various Ustilaginaceae on glucose and glycerol. Malate concentrations and itatartarate UV area after 120 h or 384 h System Duetz® cultivations in screening medium with glucose or glycerol, respectively. U. maydis MB215 ΔUmag_ria1 was used as a negative control. Error bars indicate standard deviation from the mean (n = 3). [file 40694_2018_58_MOESM1_ESM.tif]

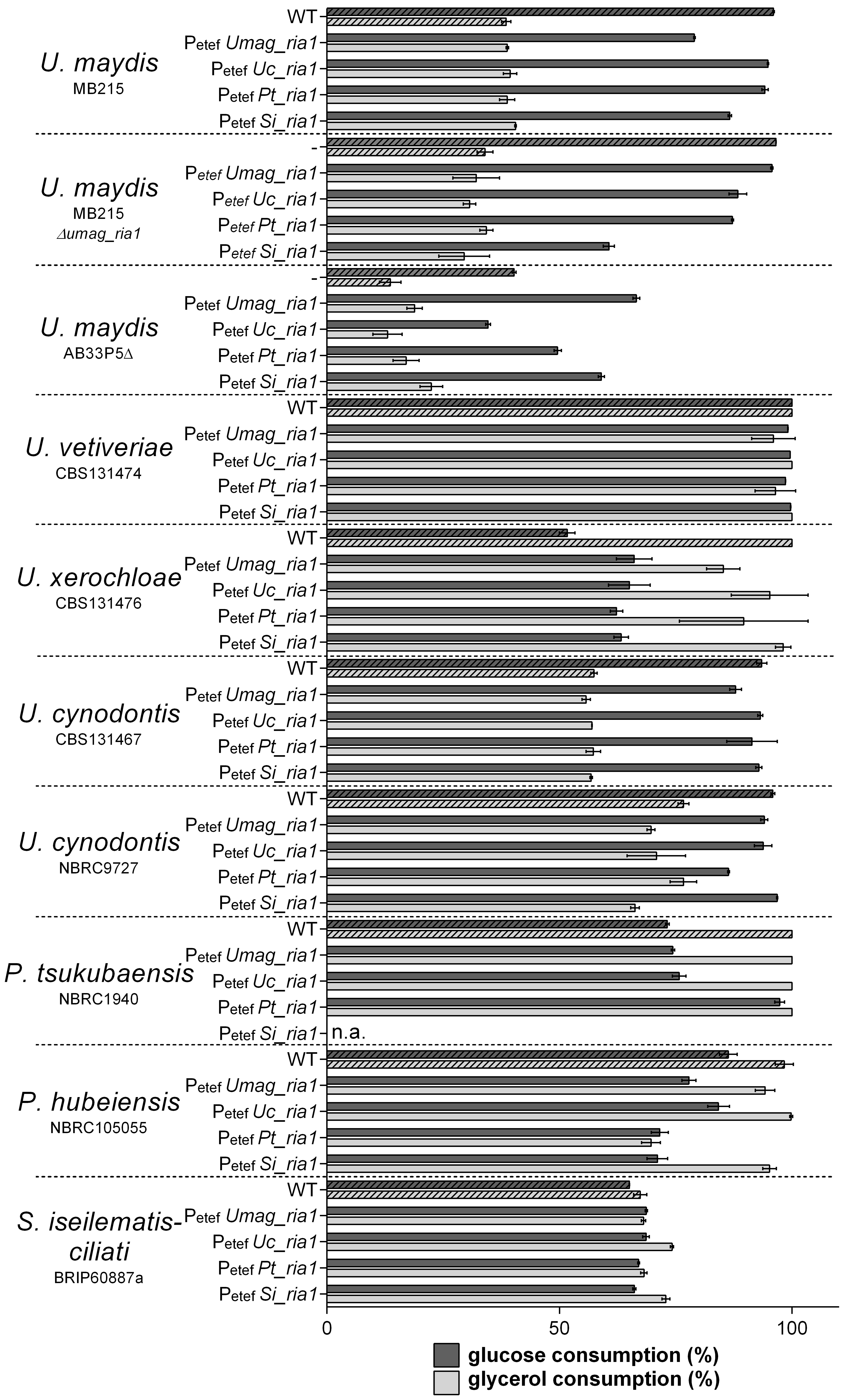

Supplement: Supplementary file 2 — Additional file 2: Fig. S2. Glucose and glycerol consumption by various Ustilaginaceae. Glucose and glycerol consumption in % after 120 h or 384 h System Duetz® cultivations in screening medium with glucose or glycerol, respectively. Error bars indicate standard deviation from the mean (n = 3). [file 40694_2018_58_MOESM2_ESM.tif]

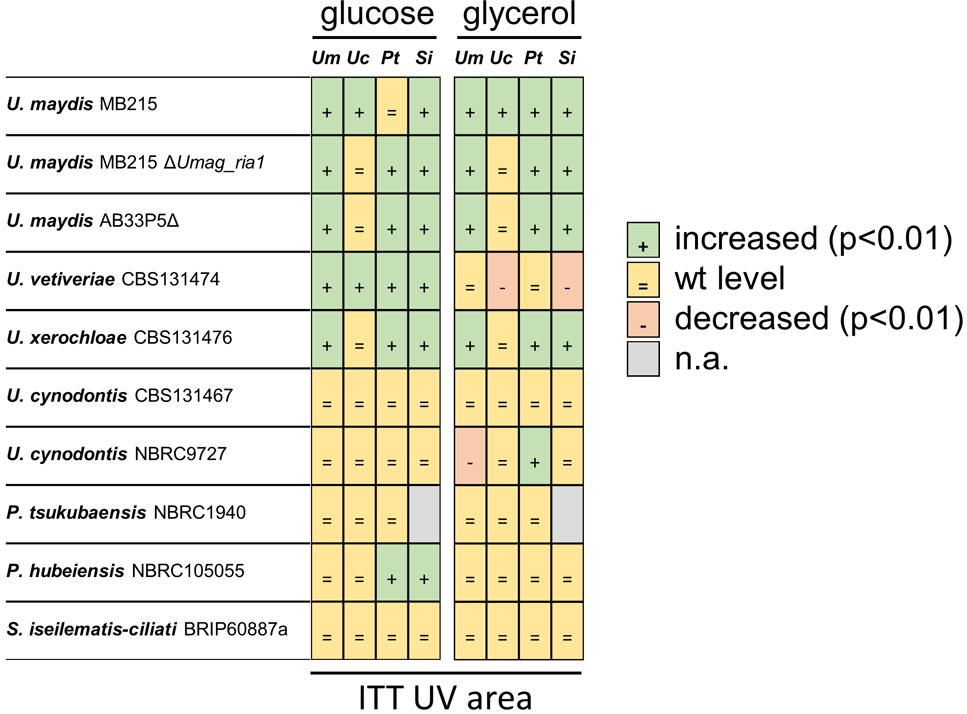

Supplement: Supplementary file 3 — Additional file 3: Fig. S3. Schematic overview of the influence of overexpression of Umag_ria1, Uc_ria1, Pt_ria1 and Si_ria1, on itatartarate (ITT) production. Differences in itatartarate production were determined after 120 h or 384 h System Duetz® cultivations in screening medium containing glucose or glycerol, respectively. [file 40694_2018_58_MOESM3_ESM.tif]

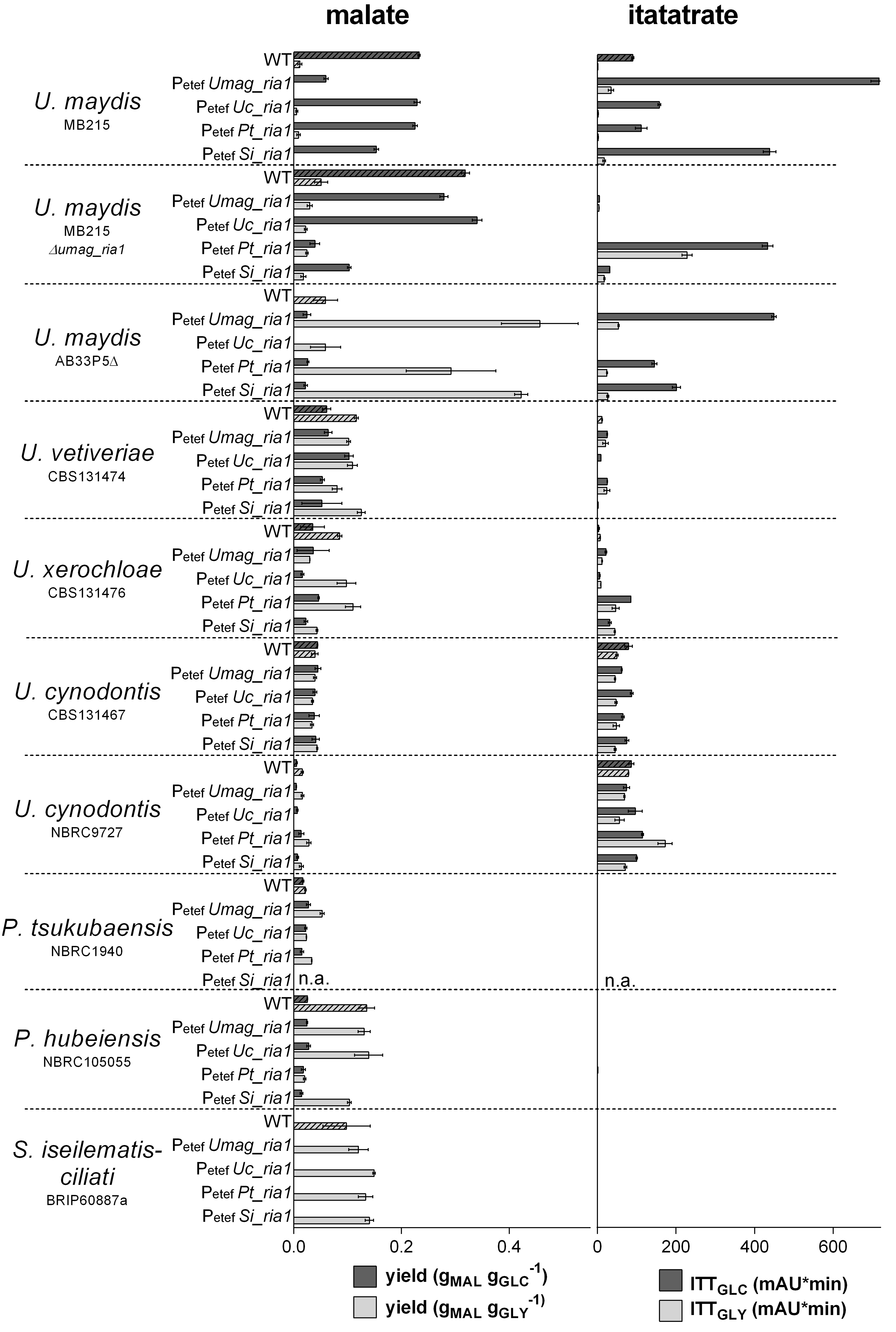

Supplement: Supplementary file 4 — Additional file 4: Fig. S4. Malate and itatartarate production by various Ustilaginaceae and their mutants transformed with Umag_ria1, Uc_ria1, Pt_ria1, Si_ria1. Malate (gMal gGLC−1, gITA gGLY−1) yield and itatartarate titer after 120 h or 384 h System Duetz® cultivations in screening medium containing glucose (GLC) and glycerol (GLY), respectively. A dash (–) indicates the negative control without an overexpression construct. Error bars indicate standard deviation from the mean (n = 3). [file 40694_2018_58_MOESM4_ESM.tif]
