# Supplementary material for: Evolutionary freedom in the regulation of the conserved itaconate cluster by Ria1 in related Ustilaginaceae
Source: Fungal Biol Biotechnol. 2018 Jul 28;5:14. doi: 10.1186/s40694-018-0058-1 (PMC6064134; doi:10.1186/s40694-018-0058-1)
Supplement: Supplementary file 5 — Additional file 5: Table S1. Oligonucleotides used for overexpression constructs. [file 40694_2018_58_MOESM5_ESM.docx]

**Additional file 5: Table S1.** Oligonucleotides used for overexpression constructs.

| **Primer name** | **Sequence (5’-3’)** |
| --- | --- |
| HT-212 | GGATCCCGTGGATGATGTTG |
| HT-213 | TCTAGAGCGGCCGCCCGG |
| HT-214 | CAACATCATCCACGGGATCCATGAGCCTCTCGAACAGCAATC |
| HT-215 | AGCCGGGCGGCCGCTCTAGATCATCGGTGCCGTCTCCTG |
| HT-216 | CAACATCATCCACGGGATCCATGAGCGTGTCAAACAGC |
| HT-217 | AGCCGGGCGGCCGCTCTAGATCATCGGTAACGCCTCTTG |
| HT-218 | CAACATCATCCACGGGATCCATGAAGATTCTCATCGACC |
| HT-219 | AGCCGGGCGGCCGCTCTAGATCAACGATGACGTTTCTTTG |
| Potef-fwd | CCAATAAAGGGCGCTGTCTC |
| Tnos-rev | CAAGACCGGCAACAGGATTC |
